# Supplementary material for: Nonequilibrium ordering dynamics of confined soft alginate hydrogel colloids driven by time-evolving electrostatic interactions
Source: Nat Commun. 2026 Mar 9;17:3662. doi: 10.1038/s41467-026-70266-w (PMC13100200; doi:10.1038/s41467-026-70266-w)
Supplement: Supplementary file 1 — Supplementary Information [file 41467_2026_70266_MOESM1_ESM.pdf]

Supplementary Information

Nonequilibrium ordering dynamics of confined soft alginate hydrogel colloids driven by time-evolving electrostatic interactions

In Hwan Jung[1†], Chetan C. Revadekar[1†], Hag Sung Lee [1], Hyerim Hwang[2,3,*], Hyosung An[4,*], and Bum Jun Park[1,*]

[1]*Department of Chemical Engineering (BK21 FOUR Integrated Engineering Program), College of Engineering, Kyung Hee University, Yongin, Gyeonggi-do 17104, South Korea*

[2]*Department of Chemical Engineering and Materials Science, Ewha Womans University, Seodaemun-gu, Seoul 03760, South Korea*

[3]*Institute for Multiscale Matter and Systems (IMMS), Ewha Womans University, Seoul 03760, Republic of Korea*

[4]*Department of Petrochemical Materials Engineering, Chonnam National University, Yeosu, Jeollanam-do 59631, South Korea*

[*]Corresponding authors:

Hyerim Hwang: hyerimhwang@ewha.ac.kr (https://orcid.org/0000-0001-8098-6030)

Hyosung An: hyosungan@jnu.ac.kr (https://orcid.org/0000-0001-8710-1012)

Bum Jun Park: bjpark@khu.ac.kr (https://orcid.org/0000-0002-5567-3523)

[†]These authors contributed equally to this work.

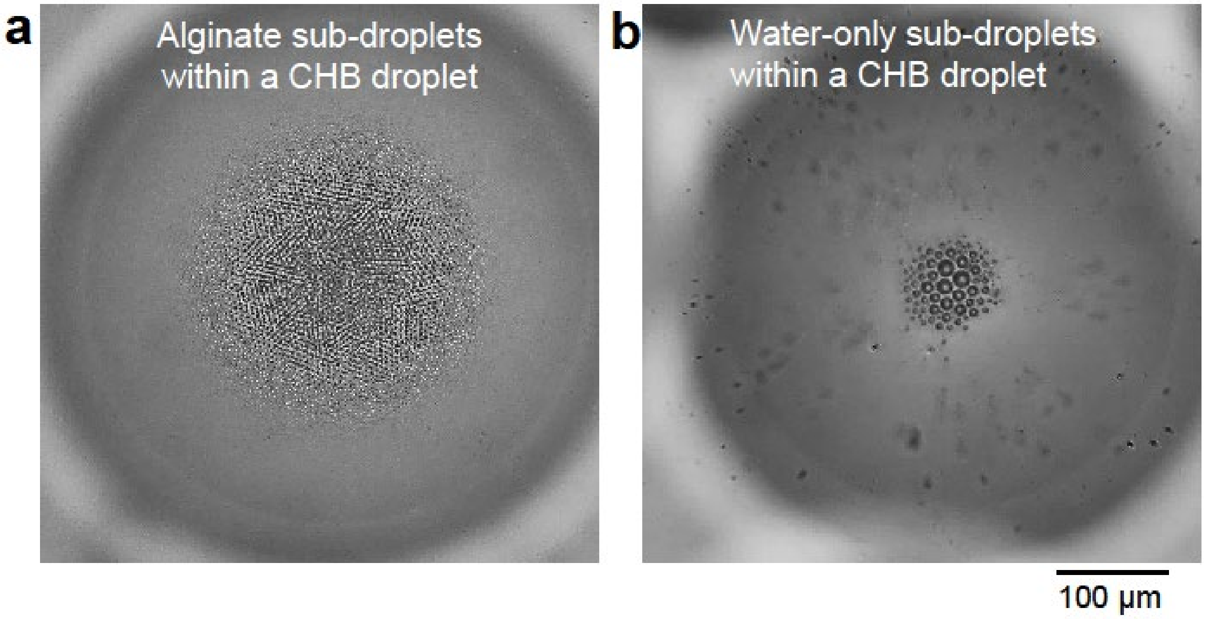

Supplementary Fig. 1 Control comparison of ordering with and without alginate inside CHB droplets. **a** Bright-field image of a CHB droplet containing alginate sub-droplets after 12 h in an external 40 mM $Ba(Ac)_2$ solution. **b** Under identical conditions, water-only sub-droplets inside a CHB droplet do not develop hexagonal order.

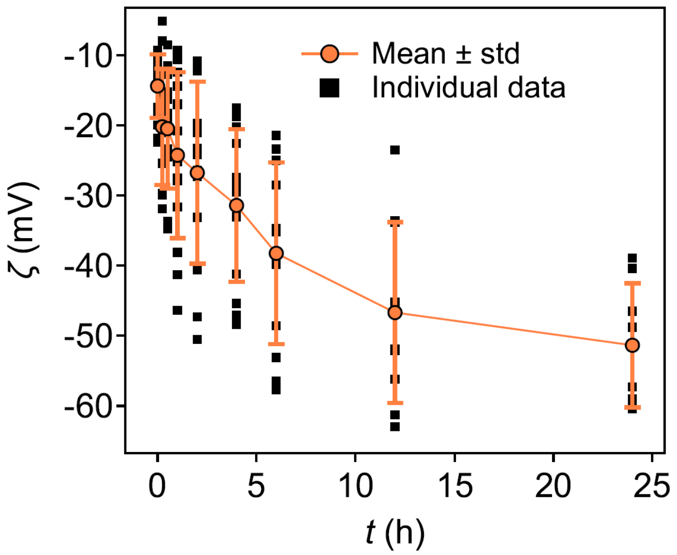

Supplementary Fig. 2 Time evolution of ζ-potential for alginate hydrogel particles in CHB. Mean ζ values plotted together with individual measurements. Error bars denote standard deviations from ≥8 independent measurements. Source data are provided as a Source Data file.

Supplementary Note 1. Interpretation of the time-dependent increase in $|\zeta|$ during Ba^{2+}-mediated hydrogelation

The evolution of the zeta potential (ζ) during Ba^{2+}-mediated hydrogelation requires careful interpretation. From a purely stoichiometric perspective, divalent-ion crosslinking bridges carboxylate groups (COO^-) within junction zones, which could reduce the number of free charges. However, the experimentally measured ζ does not directly reflect the total number of charged groups inside the hydrogel network. Instead, ζ represents the effective electrostatic potential at the shear plane and is governed by the interfacial charge density and counterion distribution near the particle–medium interface. In our system, alginate droplets initially contain Na^+ as counterions. Upon exposure to Ba^{2+}, ion exchange ($Na^+ \rightarrow Ba^{2+}$) occurs concurrently with progressive hydrogelation. This process alters the counterion environment and the electrostatic structure at the interface. As the gel network matures, restructuring and partial compaction of the hydrogel can modify the effective surface charge density. Consequently, the magnitude of ζ may increase during gelation despite local crosslink formation. Consistent with this interpretation, the measured $|\zeta|$ increases with incubation time (Supplementary Fig. 2). In parallel, partially gelled alginate droplets at early times exhibit coalescence, whereas fully incubated samples no longer show coalescence (Supplementary Fig. 10), indicating progressive stabilization associated with strengthened electrostatic repulsion.

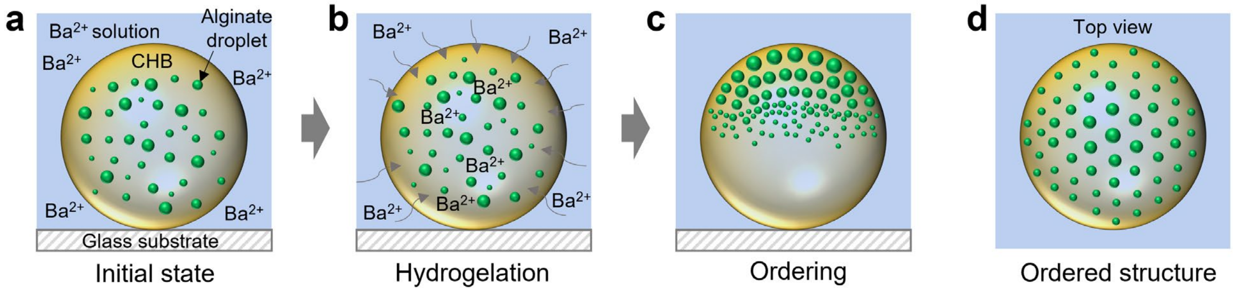

Supplementary Fig. 3 Schematic of alginate colloidal assemblies (not to scale). a Initial random dispersion in CHB. **b** Hydrogelation via Ba^{2+} influx. **c** Buoyancy-driven, size-selective flotation forming quasi-2D layers. **d** Top-view of the hexagonal arrangement.

Supplementary Note 2. Buoyancy-driven stratification and its timescale

To substantiate the buoyancy-driven stratification, we estimated the rising velocity of alginate aqueous droplets dispersed in CHB using Stokes' law. Taking a representative density difference of $\Delta\rho \approx 320$ kg m^{-3} (CHB ≈ 1.33 g cm^{-3}; 1 wt.% alginate aqueous phase ≈ 1 g cm^{-3}) and a CHB viscosity of $\eta \approx 1.5 \times 10^{-3}$ Pa s, the terminal rising velocity is given by $v = \frac{2}{9}\frac{\Delta\rho g R^2}{\eta}$, where g is the gravitational acceleration. For droplet diameters in the approximate range $2R \approx 0.5$–5 µm, the calculated velocities are on the order of ~0.01–1 µm s^{-1}. The corresponding time required to traverse the full 400 µm CHB droplet is therefore on the order of ~10^2–10^4 s (i.e., minutes to a few hours). These estimates indicate that buoyancy-driven stratification occurs on a timescale significantly shorter than the ≈6–8 h required for the emergence of pronounced hexagonal ordering. This interpretation is consistent with the experimental observations in Fig. 2c, where larger droplets are already concentrated near the upper region of the CHB droplet at early times, prior to the development of long-range hexagonal order. We therefore conclude that stratification and ordering occur on distinct timescales. Buoyancy rapidly produces quasi-2D layering, whereas the delayed onset of hexagonal ordering likely reflects the time-dependent strengthening of electrostatic interactions during Ba^{2+}-mediated hydrogelation.

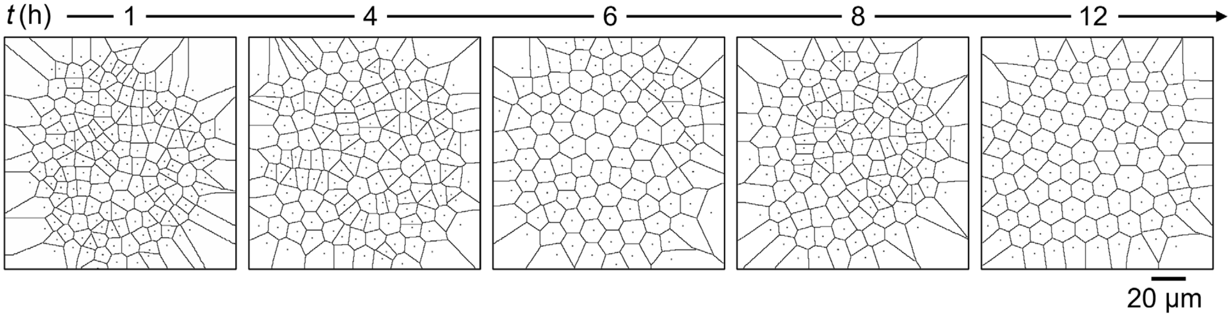

Supplementary Fig. 4 Time-resolved Voronoi tessellations of the top layer during formation of ordered alginate colloidal assemblies. Voronoi diagrams computed from particle centroids (black dots) at incubation times t = 1, 4, 6, 8, and 12 h, corresponding to the optical images in Fig. 2c.

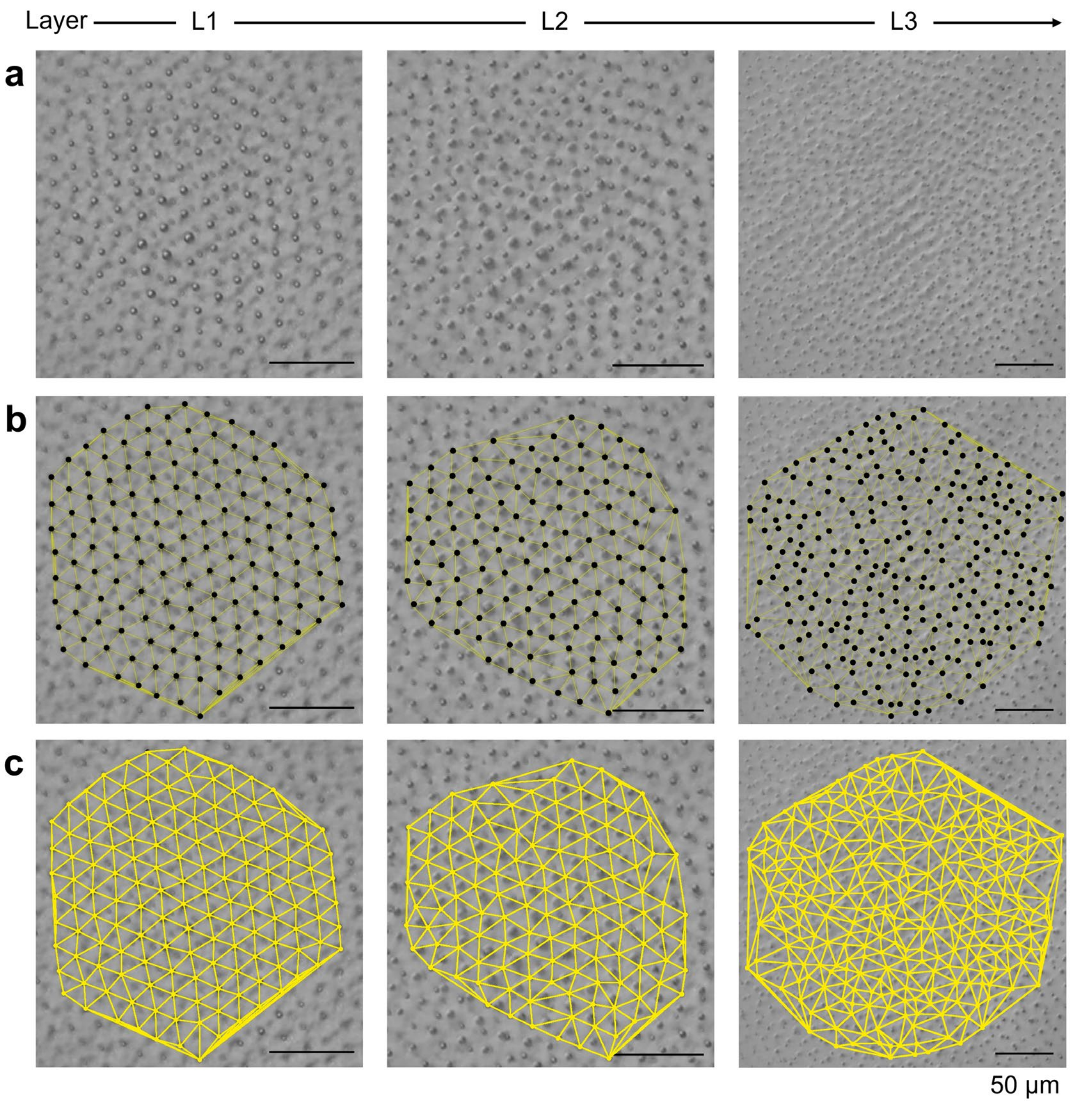

Supplementary Fig. 5 Layer-resolved imaging and triangulation of multilayered alginate colloidal assemblies. **a** Bright-field images of L1–L3 in the CHB phase. **b** Particle centroids overlaid (black dots). **c** Delaunay triangulations (yellow) highlighting local order and defects within each layer. Source data are provided as a Source Data file.

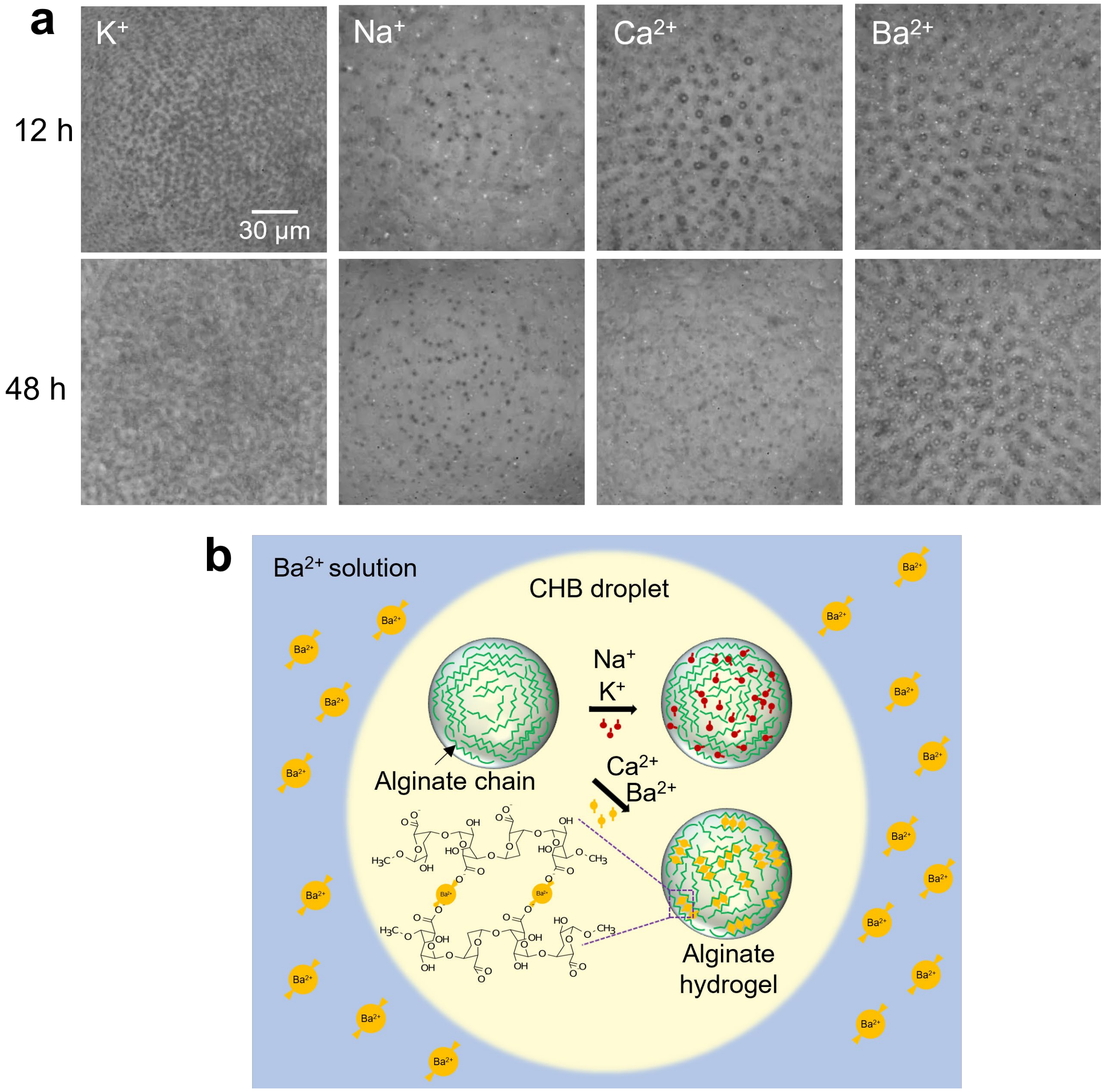

Supplementary Fig. 6 Ion-specific hydrogelation and ordering. a Bright-field images of droplets after 12 and 48 h in 40 mM monovalent (K^+, Na^+) or divalent (Ca^{2+}, Ba^{2+}) ion solutions. **b** Schematic of divalent-ion-mediated hydrogelation inside a CHB droplet.

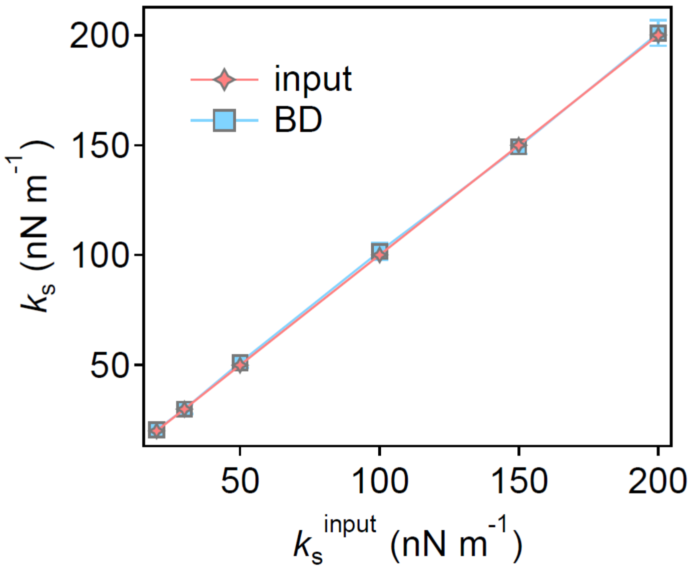

Supplementary Fig. 7 Validation of the spring-constant estimator. For each prescribed input stiffness k_s^{input} = 20–200 nN m^{-1}, blue squares show the mean over ten independent runs. Error bars denote the standard deviation and are mostly smaller than the symbol size. Source data are provided as a Source Data file.

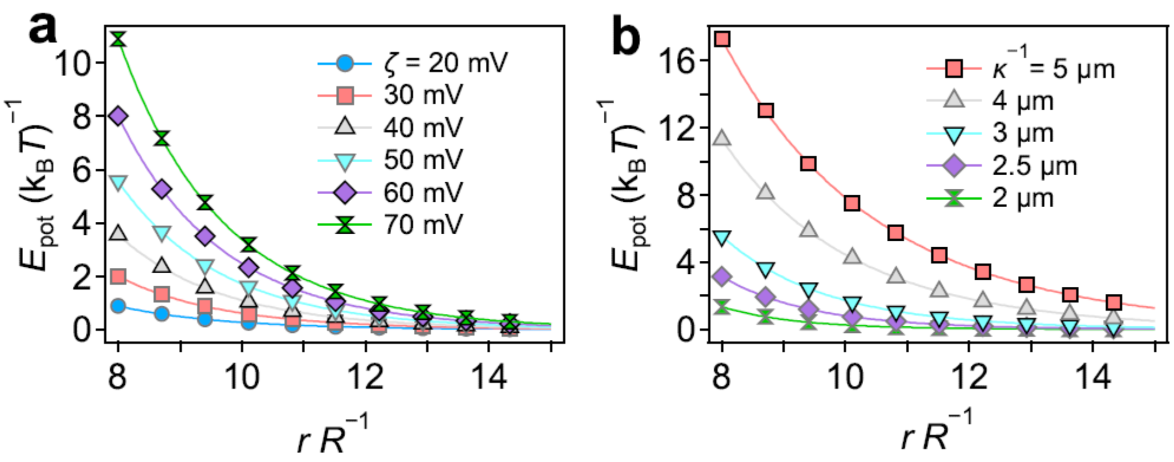

Supplementary Fig. 8 Yukawa pair potentials for BD simulations. Dimensionless pair energy $E_{\mathrm{pot}}\ (\mathrm{k_B}T)^{-1}$ as a function of normalized center–center separation $r\,R^{-1}$. **a** Curves for fixed screening length κ^{-1} = 3 µm and varying zeta potentials. **b** Curves for fixed ζ = 50 mV and varying screening length. Source data are provided as a Source Data file.

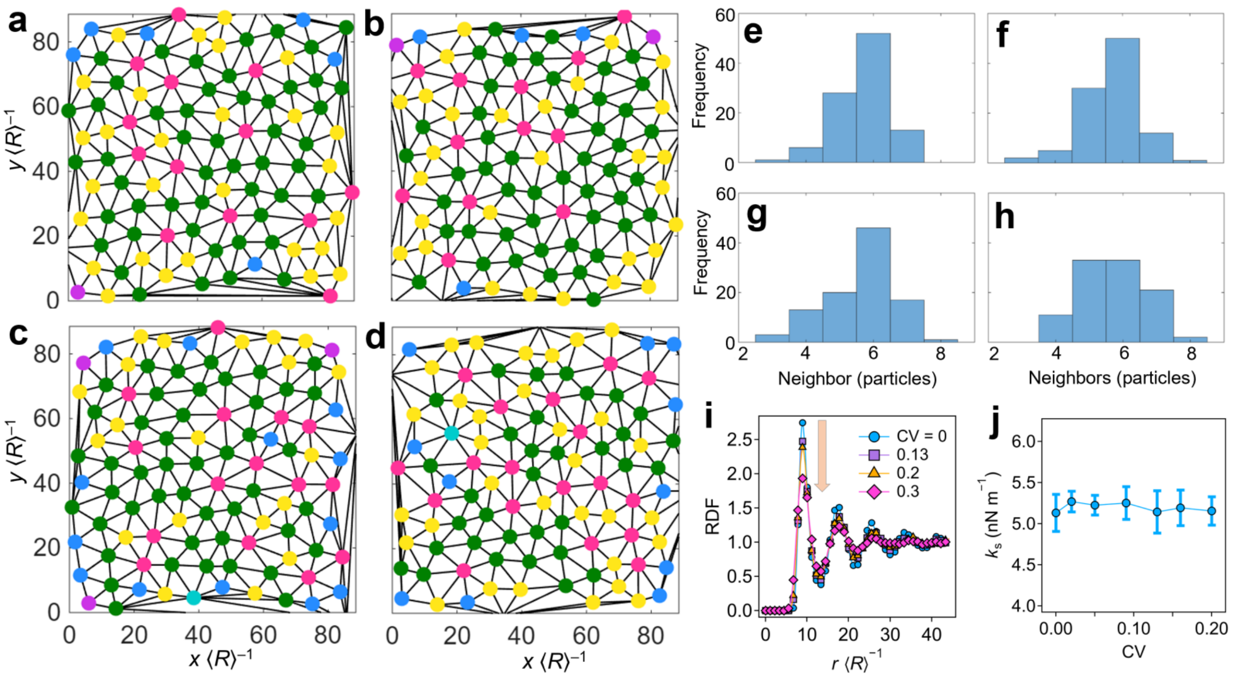

Supplementary Fig. 9 Effects of particle-size dispersity on microstructure and k_s in BD simulations. a-d Delaunay triangulations of simulated lattices with varying CV = 0 (**a**), 0.13 (**b**), 0.2 (**c**), 0.3 (**d**). Colors indicate local coordination numbers. **e-h** Corresponding histograms of the coordination number. **i** Plot of RDF versus $r\,\langle R\rangle^{-1}$. **j** Plot of k_s^{BD} versus CV. Error bars represent the standard deviation over ten independent BD runs. Source data are provided as a Source Data file.

Supplementary Note 3. Effects of particle-size dispersity on microstructure and lattice spring constant in BD simulations

As shown in Supplementary Fig. 9a–h, Delaunay triangulations and coordination-number histograms show that increasing CV progressively reduces the fraction of sixfold-coordinated sites, giving rise to defect-rich, melted-like configurations. This trend is consistent with our earlier Monte Carlo results, demonstrating that interaction heterogeneity promotes melting and destabilizes long-range order.[1, 2] Likewise, the radial distribution function (RDF) becomes increasingly attenuated and broadened with larger CV, reflecting a clear loss of long-range order (Supplementary Fig. 9i). In contrast to the strong microstructural degradation, the effective lattice spring constant k_s shows virtually no dependence on CV across the range studied. The mean values from ten independent runs overlap substantially (Supplementary Fig. 9j), indicating that the averaged local confining stiffness is relatively insensitive to particle-size dispersity despite pronounced structural disorder. These results support the use of k_s as a reliable measure of the interaction scale in our colloidal assemblies and validate the interpretation of Fig. 5 in the main text.

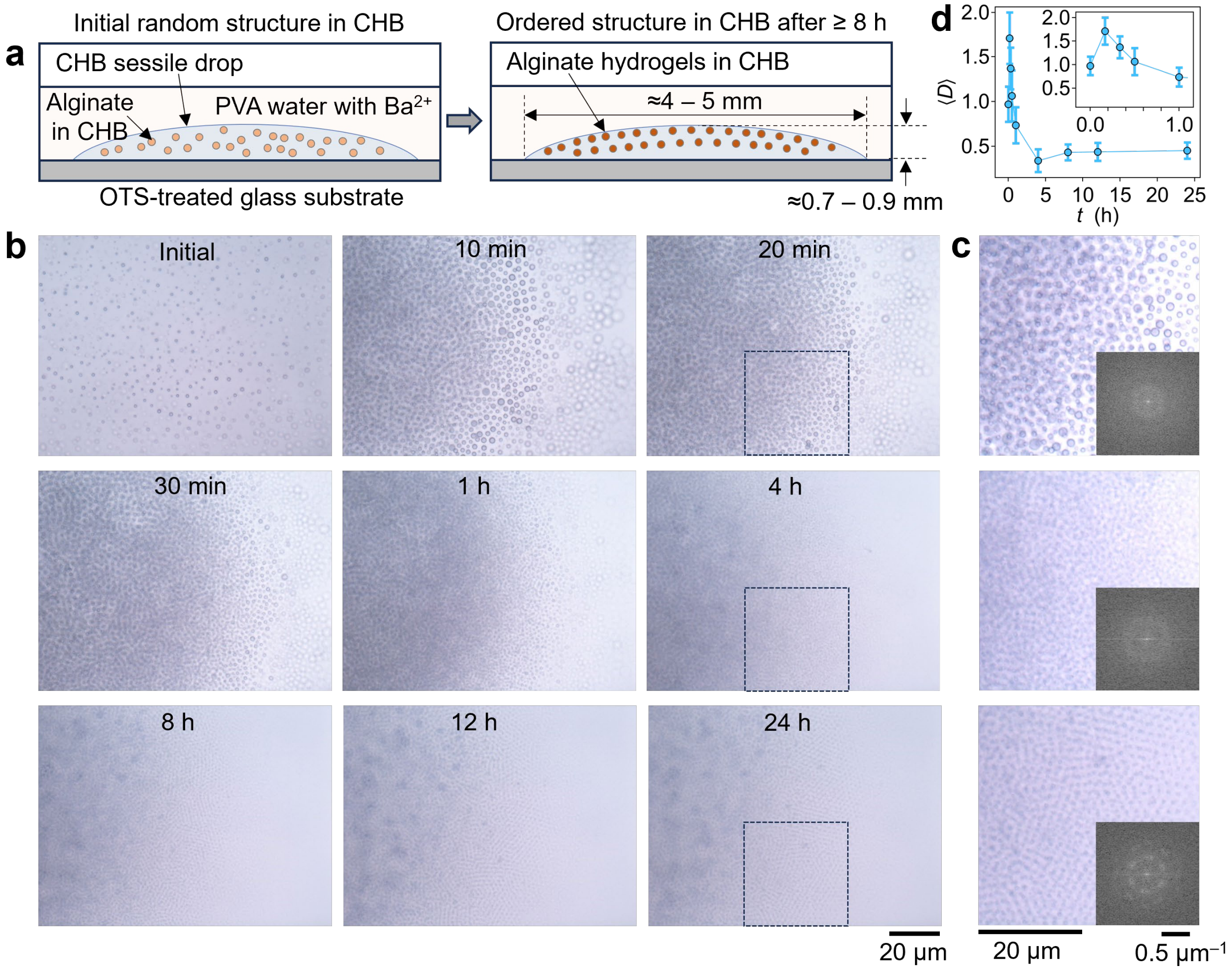

Supplementary Fig. 10 Ordering in a flattened sessile drop. a Schematic of the setup: alginate droplets in CHB confined beneath an aqueous Ba^{2+}/PVA layer. **b** Time-resolved bright-field images of ordering at the drop edge. **c** Enlarged and contrast-enhanced views of the boxed regions in panel **b**, with FFT insets. **d** Temporal evolution of mean droplet size. Inset shows the early-time regime (0–1 h). The error bars represent the standard deviations derived from 50 particles. Source data are provided as a Source Data file.

Supplementary References

1. Choi, K. H., et al. Interpretation of electrostatic self-potential measurements using interface-trapped microspheres with surface heterogeneity. *ACS Appl. Polym. Mater.* **2**, 1304-1311 (2020)

2. Park, B. J., Vermant, J. & Furst, E. M. Heterogeneity of the electrostatic repulsion between colloids at the oil–water interface. *Soft Matter* **6**, 5327-5333 (2010)
