# Supplementary material for: Nonequilibrium ordering dynamics of confined soft alginate hydrogel colloids driven by time-evolving electrostatic interactions
Source: Nat Commun. 2026 Mar 9;17:3662. doi: 10.1038/s41467-026-70266-w (PMC13100200; doi:10.1038/s41467-026-70266-w)
Supplement: Supplementary file 2 — Description of Additional Supplementary Files [file 41467_2026_70266_MOESM2_ESM.pdf]

Description of Additional Supplementary Files

Supplementary Movie 1: Bright field video corresponding to Fig. 5a, recorded at 30 fps.

Supplementary Movie 2: Bright field video corresponding to the 24 h sample shown in

Supplementary Data 1: Unprocessed raw data Excel file for Figs. 2e, 3d, 4e, 5b, and Supplementary Figs. 2, 7, 9j, 10d. (File name: Unprocessed raw data.xlsx)

Supplementary Data 2 : Custom MATLAB scripts used for BD simulations without considering particle-size dispersity. (File name: main.m)
Custom MATLAB scripts used for BD simulations with considering particle-size dispersity. (File name: main_polydispersity.m)
